# Establishment of a γ-H2AX foci-based assay to determine biological dose of radon to red bone marrow in rats

**DOI:** 10.1038/srep30018

**Published:** 2016-07-22

**Authors:** Jing Wang, Linfeng He, Dunhuang Fan, Defang Ding, Xufei Wang, Yun Gao, Xuxia Zhang, Qiang Li, Honghong Chen

**Affiliations:** 1 Department of Radiation Biology, Institute of Radiation Medicine, Fudan University, 200032 Shanghai, China; 2 Department of Radiology, Ningbo Medical Center, Lihuili Eastern Hospital, Ningbo 315100, Zhejiang, China; 3 Division of Ionizing Radiation Measurement Technology, Shanghai Institute of Measurement and Testing Technology, 201203 Shanghai, China; 4 Department of Radiological Health, Institute of Radiation Medicine, Fudan University, 200032 Shanghai, China; 5 Institute of Modern Physics, Department of Nuclear Science and Technology, Fudan University, 200433 Shanghai, China

## Abstract

The biodosimetric information is critical for assessment of cancer risk in populations exposed to high radon. However, no tools are available for biological dose estimation following radon exposure. Here, we established a γ-H2AX foci-based assay to determine biological dose to red bone marrow (RBM) in radon-inhaled rats. After 1–3 h of *in vitro* radon exposure, a specific pattern of γ-H2AX foci, linear tracks with individual p-ATM and p-DNA-PKcs foci, was observed, and the yield of γ-H2AX foci and its linear tracks displayed a linear dose-response manner in both rat peripheral blood lymphocytes (PBLs) and bone-marrow lymphocytes (BMLs). When the cumulative doses of radon inhaled by rats reached 14, 30 and 60 working level months (WLM), the yields of three types of foci markedly increased in both PBLs and BMLs, and γ-H2AX foci-based dose estimates to RBM were 0.97, 2.06 and 3.94 mGy, respectively. Notably, BMLs displayed a more profound increase of three types of foci than PBLs, and the absorbed dose ratio between BMLs and PBLs was similar between rats exposed to 30 and 60 WLM of radon. Taken together, γ-H2AX foci quantitation in PBLs is able to estimate RBM-absorbed doses with the dose-response curve of γ-H2AX foci after *in vitro* radon exposure and the ratio of RBM- to PBL-absorbed doses in rats following radon exposure.

The risk of leukemia, especially childhood leukemia induced by radon exposure, has caused great concern worldwide except for an increased risk of lung cancer induced by radon exposure^1^,^2^,^3^,^4^, although different types of radon therapy including speleotherapy and balneotherapy have long been established to treat rheumatic diseases, respiratory diseases and ankylosing spondylitis in many countries of the world^5^,^6^,^7^,^8^. Further, the efficiency of radon therapy has not been verified extensively and may be related to low radiation dose of radon exposure. Most recently, an international cohort study of radiation-monitored workers provides strong evidence for positive associations between protracted low-dose ionizing radiation (IR) exposure (mean 1.1 mGy per year) and human leukemia^9^. This finding further emphasizes the importance of dosimetric information and adherence to the basic principles of radiation protection. However, to date no effective solution is available for estimation of biological dose of organs and tissues including lung and red bone marrow (RBM) following radon and its progeny exposure because of non-uniform and low-dose exposure at several mGy levels of radon and its progeny to the human body. Although the physical dose calculation of radon and its progeny to organs and tissues has been established using International Commission on Radiological Protection (ICRP) biokinetic and dosimetric models^10^,^11^, there are large difficulties in developing reliable estimates of underground radon exposure for epidemiological studies of miners^12^,^13^. It has been recognized that the biodosimetric information is of great importance for assessment of cancer risk in populations exposed to IR not only because biodosimeters based on biomarkers can reflect the radiation damage and radiosensitivity of exposed individuals but also it may be the only means of estimating dose in the case where details of events are poorly known and no physical dose measurements are available^14^. The dicentric chromosomal assay in human peripheral blood lymphocytes (PBLs) has been currently considered as ‘gold standard’ in biological dosimetry and widely used for estimating the biological dose of whole and partial-body external radiation exposures^14^. However, its sensitivity is not sufficient for individual biological dosimetry of population receiving the radiation dose of <50 mSv high-linear energy transfer (LET)^14^. Moreover, current available biological dosimetry including cytogenetic dosimetry and other bioindicators is hard to estimate the biological dose of organs and tissues following the intake of radionuclides because of their uneven distribution in the human body^14^.

As a gas, radon mainly enters the human body through the respiratory tract and is transported to any tissue through the blood circulation. Notably, radon and its progeny distribute unevenly throughout the body due to their varying physico-chemical properties and metabolic pathways. In addition to lung, RBM is another important target organ of radon based on a higher tissue/blood partition coefficient compared to other organs^15^,^16^; moreover, the long-life progeny of radon such as ^210^Po, which is among the bone-seeking radionuclides, can accumulate in the bone over the long term, thus producing sustained high-LET α irradiation of bone-marrow hemopoietic stem cells and contributing to the development of leukemia^17^. It has been found that indoor radon at high concentrations can markedly induce DNA damage and high frequencies of micronuclei in human PBLs^18^. Moreover, Smerhovsky *et al.*^19^ have found that the frequencies of chromatid breaks and cells with chromosomal aberrations in PBLs are significantly positively correlated with the incidence of lung cancer in uranium miners. Since internal incorporation of radon and its progeny constitutes a special type of protracted irradiation, the biological dose estimation of exposure to radon and its progeny has become a difficult problem to be solved.

As a molecular marker of DNA double-strand breaks (DSBs), phosphorylated histone variant H2AX (also known as γ-H2AX) has been proven to be a critical regulatory factor that recruits various DNA repair factors to rapidly localize at DSBs and form immunostainable IR-induced foci^20^,^21^. Ataxia telangiectasia mutated (ATM) and DNA-dependent protein kinase catalytic subunit (DNA-PKcs) are the major kinases for H2AX phosphorylation^22^, which jointly regulates the repair of DSBs through non-homologous end-joining (NHEJ) and homologous recombination (HR). In recent years, the *in vitro* study of human peripheral blood and animal studies using rhesus monkey and miniature pig models have demonstrated that γ-H2AX foci assay can be used for biological dose estimation of even external irradiation and local irradiation (low-LET)^23^,^24^,^25^,^26^,^27^,^28^,^29^,^30^,^31^, and is currently the most sensitive method of biological dose estimation with a lowest dose estimate of 1 mGy^32^. Moreover, this assay has been validated through interlaboratory comparison^33^. Additionally, several laboratories have developed methods to automatically count γ-H2AX foci for application in the rapid screening of high-throughput populations^34^,^35^. On the other hand, it has been established that high-LET radiation such as α particles and heavy ions results in highly diverse and complex DSB-clustered damage, which is more difficult to repair than low-LET radiation-induced isolated-site DSBs^36^,^37^,^38^. It has been found that the kinetics of γ-H2AX foci elimination is slower after high-LET heavy ion irradiation^38^,^39^,^40^. We recently reported that the elimination rate of γ-H2AX foci induced by high-LET α-particle radiation is markedly lower than that induced by γ-ray irradiation in human PBLs^41^. Moreover, γ-H2AX foci show a specific pattern of linear track in the cell nucleus after high-LET irradiation, whereas γ-H2AX foci induced by low-LET radiation display a random, scattered distribution^38^,^41^,^42^,^43^,^44^. Therefore, the unique features of γ-H2AX foci provide an opportunity for biological dose estimation of internal irradiation of α particles for low-dose exposure to radon and its progeny.

In an effort to mimic human *in vivo* exposure to radon, a rat model suitable for γ-H2AX foci-based biodosimeter studies has been established by our laboratory based on virtually identical response of γ-H2AX foci between rat and human PBLs exposed γ-ray irradiation^45^. Based on that model, this study used *in vitro* radon exposure of rat PBLs and bone-marrow lymphocytes (BMLs) to observe the morphological features and dose-response of γ-H2AX foci and the activation of ATM and DNA-PKcs. A dose-response curve of γ-H2AX foci for *in vitro* radon exposure was established and then used to estimate RBM- and PBL- absorbed doses and their ratio in rats inhaling radon and its progeny. To determine the feasibility of the proposed method, the estimates were compared with physical doses to RBM reported in the literature^46^. Our findings indicate that pattern and quantitation of γ-H2AX foci may be used as a biomarker for determining the inhalation and estimating the biological doses to RBM and PBLs of radon and its progeny in human body.

## Results

### Formation of linear γ-H2AX foci tracks and γ-H2AX foci in rat PBLs and BMLs induced by radon exposure *in vitro*

**Figure 1:**
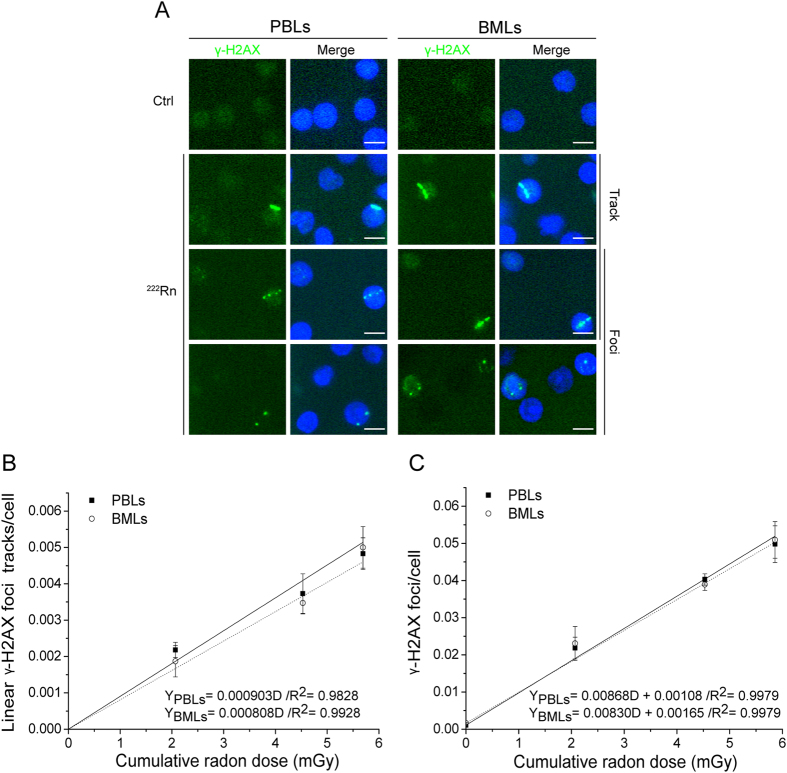
Formation of linear γ-H2AX foci tracks and γ-H2AX foci in rat PBLs and BMLs induced by radon exposure *in vitro.* (**A**) Representative image showing linear γ-H2AX foci tracks and γ-H2AX foci in PBLs and BMLs induced by radon exposure *in vitro*. (**B**) The dose-response relationship for linear γ-H2AX foci tracks in PBLs and BMLs induced by *in vitro* radon exposure for 1–3 h. (**C**) The dose-response relationship for γ-H2AX foci in PBLs and BMLs induced by *in vitro* radon exposure for 1–3 h. Three thousand to eight thousand lymphocytes from each sample were used for linear γ-H2AX foci track and γ-H2AX foci quantitation. The data are presented as averages ± standard deviations of four-six rats. Green, γ-H2AX; blue, DNA stained with DAPI. 1000 × magnification; scale bar, 5 μm.

Figure 1A shows that 1–3 h of radon exposure *in vitro* induced the formation of individual γ-H2AX foci in rat PBLs and BMLs; in addition, it resulted in continuous and discontinuous linear tracks of γ-H2AX foci. The yields of the γ-H2AX foci and its linear tracks were significantly increased with increasing cumulative radon doses, showing a linear dose-response relationship within the dose range of 0–5.86 mGy (Fig. 1B,C). Moreover, the formation of linear γ-H2AX foci tracks and individual γ-H2AX foci induced by *in vitro* radon exposure showed good consistency between PBLs and BMLs. However, the yield of individual foci was roughly 10-fold higher than that of linear tracks. By comparison, fewer γ-H2AX foci were formed in cells exposed to the environmental radon background, whereas linear γ-H2AX foci tracks were not observed in the control cells. There was no significant difference between PBLs and BMLs in the control group.

### Formation of p-ATM and p-DNA-PKcs foci and their co-localization with γ-H2AX foci in rat PBLs and BMLs induced by radon exposure *in vitro*

**Figure 2:**
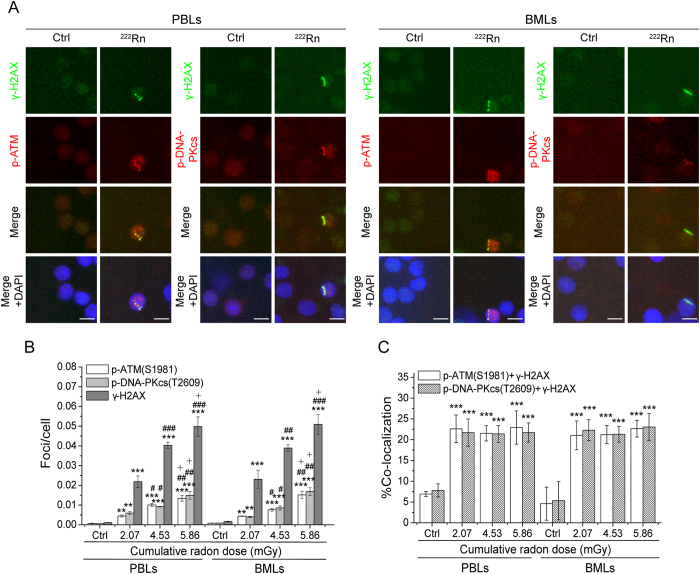
Formation of p-ATM and p-DNA-PKcs foci and their co-localization with γ-H2AX foci in rat PBLs and BMLs induced by radon exposure *in vitro*. (**A**) Representative image showing p-ATM and p-DNA-PKcs foci and their co-localization with γ-H2AX foci in PBLs and BMLs induced by radon exposure *in vitro*. (**B**) The dose-response for p-ATM and p-DNA-PKcs foci in PBLs and BMLs induced by *in vitro* radon exposure. (**C**) The co-localization ratios of p-ATM and p-DNA-PKcs foci with γ-H2AX foci in PBLs and BMLs induced by *in vitro* radon exposure. Three thousand to eight thousand lymphocytes from each sample were used for p-ATM and p-DNA-PKcs foci quantitation. The data are presented as averages ± standard deviations of four-six rats. Green, γ-H2AX; red, p-ATM and p-DNA-PKcs; blue, DNA stained with DAPI. 1000 × magnification; scale bar, 5 μm. ^*^P < 0.05, ^**^P < 0.01, ^***^P < 0.001 compared with the background value of control group for the same protein in the same type of cells, ^#^*P* < 0.05, ^##^*P* < 0.01, ^###^*P* < 0.001 compared with the same protein in the same type of cells at the dose of 2.07 mGy, and ^+^*P* < 0.05 compared with the same protein in the same type of cells at the dose of 4.53 mGy.

Figure 2A shows the formation of p-ATM and p-DNA-PKcs foci in rat PBLs and BMLs after 1–3 h of *in vitro* radon exposure. Similar morphology and distribution of p-ATM and p-DNA-PKcs foci were observed between PBLs and BMLs. Both p-ATM and p-DNA-PKcs foci were distributed along linear γ-H2AX tracks either as individual foci, scattered with γ-H2AX foci, or present alone. The numbers of p-ATM and p-DNA-PKcs foci induced by radon exposure significantly increased compared with the background values in PBLs and BMLs. The marked increase was also observed with increasing cumulative radon dose, and there was no significant difference between the two types of cells. The yield of p-ATM foci induced by radon exposure was similar to the yield of p-DNA-PKcs foci, but was remarkably lower than the yield of γ-H2AX foci (Fig. 2B). It is notable that compared with the background values, the co-localization ratios of p-ATM and p-DNA-PKcs foci with γ-H2AX foci were significantly higher in PBLs and BMLs exposed radon and its progeny. However, instead of changing substantially with increasing cumulative radon doses, the co-localization ratios fluctuated in the range of 20–25% on average, with no significant differences between different cells (Fig. 2C). In comparison, the values of spontaneous p-ATM and p-DNA-PKcs foci were relatively low in rat PBLs and BMLs exposed to the environmental radon background, and the co-localization ratios with γ-H2AX foci were substantially low. Moreover, the values of spontaneous p-ATM and p-DNA-PKcs foci showed no significant difference in the same type of cells or between two types of cells.

### γ-H2AX, p-ATM and p-DNA-PKcs foci formation and co-localization of p-ATM and p-DNA-PKcs with γ-H2AX foci in the PBLs and BMLs of rats inhaling radon and its progeny

**Figure 3:**
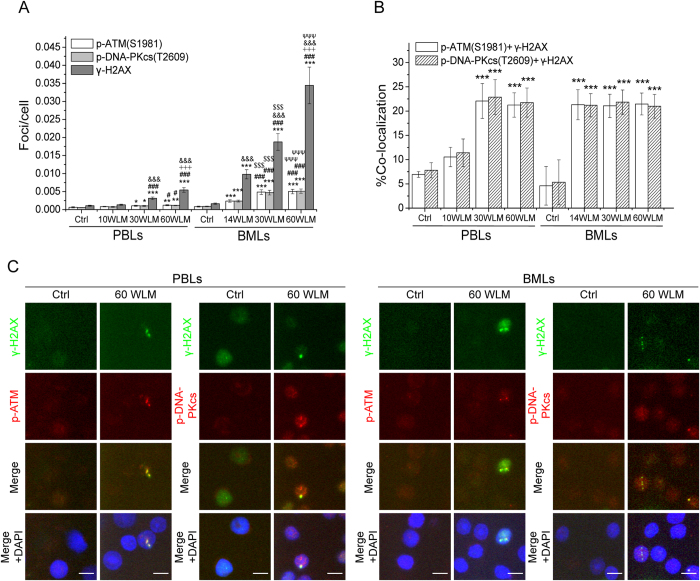
Formation of γ-H2AX, p-ATM and p-DNA-PKcs foci in PBLs and BMLs of rats inhaling radon. (**A**) The dose-response for γ-H2AX, p-ATM and p-DNA-PKcs foci in the PBLs and BMLs of rats inhaling radon. (**B**) The co-localization ratios of p-ATM and p-DNA-PKcs foci with γ-H2AX foci in the PBLs and BMLs of rats inhaling radon. (**C**) Representative images showing γ-H2AX foci and its linear tracks and co-localization of p-ATM and p-DNA-PKcs foci with γ-H2AX foci in the PBLs and BMLs of rats inhaling 60 WLM radon. Three thousand to eight thousand lymphocytes from each sample were used for γ-H2AX, p-ATM and p-DNA-PKcs foci quantitation. The data are presented as averages ± standard deviations of five-ten rats. Green, γ-H2AX; red, p-ATM and p-DNA-PKcs; blue, DNA stained with DAPI. 1000 × magnification; scale bar, 5 μm. ^*^P < 0.05, ^**^P < 0.01, ^***^P < 0.001 compared with the background value for the same protein in the same type of cells, ^#^*P* < 0.05, ^###^*P* < 0.001 compared with 10-WLM or 14-WLM radon exposure group for the same protein in the same type of cells, ^﹢﹢﹢^*P* < 0.001 compared with 30-WLM radon exposure group for the same protein in the same type of cells, ^&&&^*P* < 0.001 compared with p-ATM and p-DNA-PKcs foci under the same irradiation conditions, ^$$$^*P* < 0.001 compared with 30-WLM radon exposure group for the same protein in the PBLs, and ^ΨΨΨ^*P* < 0.001 compared with 60-WLM radon exposure group for the same protein in the PBLs.

Figure 3A shows a remarkable growth (compared with the background values) in the numbers of γ-H2AX, p-ATM and p-DNA-PKcs foci in rat PBLs and BMLs after overall inhalation of 14–60 WLM radon. Moreover, the number of three types of foci was substantially increased with increasing cumulative radon dose. The number of γ-H2AX foci was significantly higher than those of p-ATM and p-DNA-PKcs foci, whereas no significant difference was observed between the latter two in either rat PBLs or BMLs. It is worth noting that BMLs had significantly higher numbers of γ-H2AX, p-ATM and p-DNA-PKcs foci than PBLs, possibly due to the accumulation of radon and its progeny in RBM. Additionally, the co-localization ratios (21–22%) of p-ATM and p-DNA-PKcs foci with γ-H2AX foci in the PBLs and BMLs of rats inhaling 14–60 WLM radon were significantly higher than those of rats inhaling the background radon, which showed no obvious changes with increasing cumulative radon dose and was similar between PBLs and BMLs (Fig. 3B), consistent with the results from the *in vitro* cell assay. In contrast, the co-localization ratios in PBLs of rats inhaling 10 WLM radon were not higher than the background values since the numbers of three types of foci were comparable to the background values.

Morphological observation revealed that γ-H2AX, p-ATM and p-DNA-PKcs foci were primarily distributed randomly as individual foci in PBLs and BMLs. However, BMLs also showed linear γ-H2AX foci tracks in rats inhaling 60 WLM radon, a result similar to those formed in cells exposed to radon *in vitro* (Fig. 3C).

### Biological dose estimation of the PBLs and RBM in rats inhaling radon and its progeny

PBL- and BML-absorbed doses in rats inhaling radon and its progeny were estimated using the γ-H2AX foci dose-response curve of the PBLs and BMLs exposed to radon *in vitro*. Table 1 shows that PBL- and RBM-absorbed doses increased with the cumulative radon doses of 10, 14, 30 and 60 WLM, whereas absorbed doses of the two types of cells were substantially low: incalculable–0.5 mGy and 1–4 mGy, respectively. The RBM-absorbed doses were approximately 8.76 and 7.90 times those of PBLs, which could be related to the accumulation of radon and its progeny in RBM.

Sakoda *et al.*^46^ have established a physiologically based pharmacokinetic (PBPK) model of inhaled radon for mice, rats and humans and calculated the absorbed doses to their major organs and tissues including RBM. The results showed that the RBM-absorbed dose rate for rats inhaled radon was 0.532 nGy/((Bq/m^3^)•day), which was similar to that of mice and humans inhaled radon. Following from the definition that 1 WLM is the exposure to potential α energy of radon progeny from 1 month (170 h) of irradiation at 3,700 Bq/m^3^, the RBM-absorbed dose at the cumulative radon dose of 1 WLM can be estimated to be 0.0139 mGy. Likewise, RBM-absorbed doses at the cumulative radon doses of 14, 30, and 60 WLM were 0.1952, 0.4183 and 0.8366 mGy. By comparison, the estimates of RBM-absorbed doses at the three cumulative radon doses in our experiments were 4.99, 4.92 and 4.71 times that reported in the above-mentioned literature (Table 1).

### Detection of damage in PBLs and BMLs of rats inhaling radon and its progeny

Table 2 shows that the percentage of pan-nuclear γ-H2AX-positive cells in PBLs and BMLs showed no significant change with increasing cumulative radon dose in rats after overall radon inhalation of 10–60 WLM, despite an increasing trend in the 60 WLM group. Similarly, the PCE/NCE ratio had no obvious change, although the frequency of PCEMN markedly increased in the 60 WLM group.

## Discussion

In this study, similar to our previous study on human PBLs by *in vitro* irradiation with α-particles from ^241^Am^41^, a specific pattern of γ-H2AX foci, linear γ-H2AX foci tracks, was observed in rat PBLs and BMLs exposed to radon and its progeny *in vitro*. Meanwhile, ATM and DNA-PKcs were phosphorylated and activated to form the individual foci in rat PBLs and BMLs exposed *in vitro* to radon. Strikingly, the tracks of γ-H2AX foci primarily displayed a continuous linear track pattern after 1-hour radon exposure even at a substantially low dose of 2.07 mGy, whereas continuous and discontinuous linear tracks were observed with extended periods of radon exposure. It has been reported that γ-H2AX foci formed at DSB ends can extend to the intact DNA strands on both sides^20^,^21^, while p-ATM and p-DNA-PKcs foci are only located at the DSB-flanking chromatin^47^,^48^, which may be an important reason for the formation of a continuous linear γ-H2AX foci tracks and individual p-ATM and p-DNA-PKcs foci. With the extension of radon exposure time, new DSBs are continually produced, whereas old DSBs are continually repaired. In these processes, γ-H2AX may be either degraded or released, leading to the gradual emergence of a discontinuous morphology of linear tracks. Likewise, linear γ-H2AX foci tracks were also observed in the BMLs of rats inhaling 60 WLM radon. Studies have shown that the formation of linear γ-H2AX foci tracks is one of the characteristics of DSBs induced by high-LET radiation (as distinct from low-LET radiation)^49^,^50^,^51^. Taken together, our results suggest that the formation of linear γ-H2AX foci tracks and the distribution of individual p-ATM and p-DNA-PKcs foci along the linear tracks in rat PBLs and BMLs can be used as a biomarker to determine exposure to radon and its progeny when high-LET external irradiation and other internal radiation from α nuclides are excluded.

Although linear γ-H2AX foci tracks were characteristic pattern after exposure to radon, the yield of individual foci was significantly higher than that of linear tracks in rat PBLs and BMLs exposed to radon and its progeny *in vitro*. More importantly, the dose-response of PBLs and BMLs induced by *in vitro* radon exposure showed great consistency, regardless of either the individual γ-H2AX foci or its linear tracks. This finding indicates that detection of γ-H2AX foci or tracks in PBLs can directly represent the response of BMLs to radon exposure. Moreover, obvious increase of co-localization ratios of p-ATM or p-DNA-PKcs foci with γ-H2AX foci was consistent between PBLs and BMLs induced by *in vitro* radon exposure, which might be an important reason for the consistent yield of γ-H2AX foci between the two types of cells since co-localization of activated ATM or DNA-PKcs with γ-H2AX at DSBs has been found to be the key factor for the effective repair of DSBs^40^. Additionally, regardless of *in vitro* exposure of cells to radon or *in vivo* exposure of rats to radon, the co-localization ratio of p-ATM or p-DNA-PKcs foci with γ-H2AX foci in PBLs and BMLs remained constant at approximately 20–25%, suggesting that 20–25% of DSBs induced by high-LET radon were repaired through the NHEJ pathway with fixed repair efficiency. This is in agreement with the reports that high-LET irradiation induced ≤25% DSBs repair dependent on ATM^52^. Further comparison of the dose-response level between γ-H2AX and p-ATM or p-DNA-PKcs foci revealed that the yield of p-ATM foci and p-DNA-PKcs foci was similar but significantly lower than that of γ-H2AX foci in PBLs and BMLs, as induced by exposure to radon and its progeny *in vitro* and *in vivo*. It was reported that ATM participates directly in the NHEJ-mediated repair of DSBs, which facilitates DSBs repair through DNA-PKcs phosphorylation at Thr2609 and synergy with DNA-PKcs autophosphorylation^53^. This mechanism may explain our observation about the similar numbers of p-ATM foci and p-DNA-PKcs foci induced by radon exposure. Moreover, in addition to H2AX phosphorylation by activated ATM, DNA-PKcs can directly phosphorylate H2AX via a non-ATM-dependent pathway and indirectly regulate the level of H2AX phosphorylation via other signaling pathways in the DNA damage response process^54^. All of these processes may be the underlying causes of the significantly smaller numbers of p-ATM and p-DNA-PKcs foci than γ-H2AX foci induced by exposure to radon and its progeny.

In this study, PBL- and RBM-absorbed doses in rats inhaling radon and its progeny were estimated using the established dose-response curve of γ-H2AX foci for rat PBLs and BMLs exposed to radon *in vitro*. The estimates of RBM-absorbed doses in rats exposed to radon of 14, 40, and 60 WLM were 0.97, 2.06 and 3.94 mGy, respectively, which were 4.99, 4.92 and 4.71 times the physical dose calculated by Sakoda *et al.*^46^ using PBPK model^11^. The higher RBM-absorbed doses obtained in this study might be related to the higher dose rate of radon exposure in the establishment of the *in vitro* dose-response curve. From another point of view, our estimates of RBM-absorbed doses in rats exposed to radon of 14, 30 and 60 WLM were similarly higher than the values reported in the literature mentioned above, indicating that the established method of the γ-H2AX foci dose-response curve has a certain feasibility for estimating the RBM-absorbed doses. On the other hand, in terms of the allowable error in biological dose estimation of external irradiation by dicentric aberration analysis, with an irradiation dose <1 Gy, a relative deviation within 30% is considered acceptable; with an irradiation dose >1 Gy, a relative deviation within 20% is deemed qualified. Thus, considerable deviation may occur when estimating substantially low absorbed doses at several mGy levels. Further analysis of the absorbed dose ratios between RBM and PBLs in rats inhaling radon and its progeny revealed that the absorbed dose ratios between RBM and PMLs were similar in rats exposed to radon of 30 and 60 WLM, i.e., 8.76 and 7.90 times, also demonstrating the feasibility of the established γ-H2AX foci-based method for RBM-absorbed dose estimation. This finding also demonstrated that amount of radon and its progeny accumulated in RBM was much higher than that of blood.

Although the number of γ-H2AX foci was significantly higher in the PBLs and BMLs of rats inhaling radon and its progeny at 14, 30 and 60 WLM compared with the control rats, the frequency of nuclear γ-H2AX-positive cells representing apoptosis^55^,^56^,^57^ and PCE/NCE ratio showed no obvious changes, indicating that the DSBs damage could be repaired by the cells without causing increased apoptosis at RBM-absorbed doses of ≤3.94 mGy. The frequencies of PCEMN were only markedly increased at radon exposure of 60 WLM, RBM-absorbed dose of 3.94 mGy compared with the control group. It has been reported that radon exposure at a cumulative dose of 13.01–65.05 WLM induced the damaging effects of hematopoietic stem/progenitor cells in mice, showing the increases of chromosomal breaks, chromatid breaks and PCEMN frequencies and the decrease of the mitotic index of bone-marrow cells^58^. Moreover, when the cumulative radon dose inhaled by mice reached 105 WLM, bone-marrow cell apoptosis also increased, whereas granulocyte colony formation and ^3^H-TdR incorporation both declined significantly^59^. The dose-response difference between our results and those reported in the literature could be related not only to the fact that rats are larger than mice but also to the fact that the rats were exposed to lower doses of radon than mice.

In summary, exposure to radon and its progeny can induce the formation of continuous or discontinuous linear tracks of γ-H2AX foci with individual p-ATM and p-DNA-PKcs foci distributed along the linear γ-H2AX foci tracks in rat PBLs and BMLs. The linear γ-H2AX foci tracks in rat PBLs and BMLs can serve as a biomarker to determine whether the body is suffered from high radon exposure in the absence of other internal radiation from α nuclides and high-LET external irradiation. More importantly, γ-H2AX foci quantitation in PBLs can be used to estimate RBM-absorbed doses with the established linear dose-response curve of γ-H2AX foci after *in vitro* radon exposure and the established ratio of RBM- to PBLs-absorbed doses after *in vivo* radon exposure, although the reliability of this estimation remains to be further verified in radon-exposed population. Our study has established a connection of mutual authentication between biological dose and physical dose determined by RBM dose estimation under complex circumstance of uneven distribution of radionuclides in the human body. Furthermore, absorbed dose of other organs and tissues such as lung can be subsequently assessed by the physical method based on biological dose of RBM particularly when there are difficulties in detecting the uptake of radon and its progeny. Altogether, our findings are of great significance not only in the risk assessment for leukemia and lung cancer induced by radon exposure but also in the prevention and control of human health risk caused by radon exposure.

## Materials and Methods

### Animals and PBLs and BMLs isolation

The experiments with animals were approved by the Animal Research Ethics Committee of School of Pharmacy of Fudan University in accordance with China experimental animal administrative regulations. Specific-pathogen-free (SPF) male Sprague-Dawley (SD) rats, weighing 200 ± 20 g, were provided by the Experimental Animal Center of Fudan University. The rats were anesthetized with 10% chloral hydrate and disinfected with 70% alcohol. Blood specimens were collected from rat heart and transferred into heparinized tubes; bone marrow was taken from one side of the femur on a clean bench. PBLs and BMLs were isolated by density gradient centrifugation with the specific rat PBL isolation solution (Cat. No. LTS1083) and BML isolation solution (Cat. No. TBD2013LR), respectively, from Haoyang Biological Products Technology Co., Ltd., Tianjin, China, following the manufacturer’s instructions. The isolated lymphocytes were resuspended (~1 × 10^6^ cells/mL) with RPMI 1640 culture medium containing 15% fetal bovine serum (GIBCO, Invitrogen Technologies, Carlsbad, CA), 5% rat plasma, 100 U/mL penicillin and 100 mg/L streptomycin. The cell suspension was incubated at 37 °C under saturated humidity.

### The *in vitro* radon exposure system and radon exposure of isolated PBLs and BMLs *in vitro*

The *in vitro* radon exposure system was set up according to design principles described in the literature^60^. Radon gas was pumped from a ^226^Rn source of activity 135 kBq and circulated into a closed chamber with saturated humidity that permits exposure of cell monolayers cultured on 6-well Transwell membrane to radon and its progeny. The exposure chamber was placed in the water-jacketed incubator with constant temperature at 37 °C. Radon concentration was continuously monitored throughout the exposure time with RAD7 electronic radon detector controlled by a computer (Durridge Company Inc., Billerica, MA). As CO_2_ is unnecessary for the lymphocyte short-term culture *in vitro*^28^,^30^,^61^,^62^, the exposure chamber was not connected with a CO_2_ supply.

Approximately 3 × 10^6^ lymphocytes were seeded into the 0.4 μm pore polyester membrane of 6-well Transwell inserts (Corning Incorporated. Life Sciences, Lowell, MA) pre-coated overnight with a mixture of 50 mg/L poly-lysine (PLL) (Sigma, Saint Louis, MO) and 100 mg/L poly-ornithine (PLO) (Sigma) (v/v = 5:2). The Transwell plate was incubated in a cell culture incubator at 37 °C under saturated humidity for 4 h, allowing the lymphocyte monolayer to adhere a transparent polyester membrane. After cell adherence, non-adherent suspended cells were washed away using the culture medium. The volume of culture medium in the lower chamber was adjusted to just reach the film so that the cells would be kept moist. A CR-39 solid-state nuclear track detector (Fukuvi Chemical Industry Co., Ltd, Fukui, Japan) was placed in a cell free insert of 6-well Transwell plate to measure the dose absorbed from radon exposure. This Transwell plate was set into the *in vitro* radon exposure system containing radon concentration of ~1000,000 Bq/m^3^ and kept at 37 °C under saturated humidity for 1–3 h of exposure to radon. The control lymphocytes were exposed simultaneously to the environmental radon background in a cell culture incubator at 37 °C under saturated humidity for the same exposure time.

### Estimation of absorbed dose of radon exposure *in vitro*

CR-39 sheets were taken out at indicated time points of cells exposure to radon. Track density was counted after etching, as reported by Tokonami *et al.*^63^: first, five fields of view were randomly selected from each CR-39 sheet for image acquisition and counting using an optical microscope (4×)^64^; next, the track density F (tracks/μm^2^) of the CR-39 sheet was calculated based on the area of the field of view on the image acquired (2.64 mm^2^) by optical microscopy (4×). The LET value of α particles (5.49 MeV) emitted by ^222^Rn to cells was calculated to be 139 keV/μm, using the SRIM program in accordance with the geometrical parameters of the cell-exposure apparatus and the physical arrangement of the cell exposure plate. The absorbed dose of cells was obtained using the following formula:


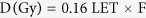


An overly high CR39 track density might not only result in difficulty achieving an accurate count but also track overlapping. To ensure the accurate counts of the tracks, the radon exposure dose was controlled to limit the particle track density of CR-39 to less than 3.8 × 10^−4^ tracks/μm^2^.

### The multifunctional ecological radon chamber and *in vivo* exposure of rats to radon

The multifunctional ecological radon chamber with 0.50 m^3^ volume was constructed according to the HD-3 multifunctional ecological radon chamber described in the literature^65^. Radon gas in one inhalation chamber was from a ^226^Rn source of activity 135 kBq, and another one was from a ^226^Rn source of activity 1980 kBq. Radon gas was introduced and circulated between ^226^Rn source and inhalation chamber. Oxygen in the inhalation chamber was monitored and introduced with oxygen generator, and the inhalation chamber had an air outlet pipeline to make the inside of chamber with the same environment without overpressure. The temperature and humidity was suitable for rats with regulating the humidity with dried CaCl_2_. To stabilize the radon concentration in the chambers before *in vivo* exposure of rats to radon, radon gas was continuously pumped into the chambers 24 hours a day for three days and then 12 hours a day for another three days with outlet pipeline open to the atmosphere. The radon concentration in the radon chambers was continuously monitored and recorded with a RAD7 electronic radon detector during the pumping. On the seventh day, the radon concentration was stabilized, and radon chambers were ready for the experiments. To maintain relatively stable dose rates from radon exposure, each cage containing a rat was introduced through the double door port and radon concentration in the radon chambers was continuously monitored and recorded using the RAD7 electronic radon detector during 12 h radon exposure.

SPF male SD rats weighing 200 ± 20 g were randomly divided into five groups by weight, including one background control and four radon-exposure groups with different doses (*n* = 5–10 each group). The cumulative doses of radon and its progeny were 10, 14, 30 and 60 working-level months (WLM). The 10 WLM and 14 WLM groups were exposed to ~40,000 Bq/m^3^ radon in the multifunctional ecological radon chamber, 6 d per week and 12 h per day, for a total exposure time of 158 and 221 h, respectively. The 30 WLM and 60 WLM groups were exposed to ~100,000 Bq/m^3^, 6 d per week and 12 h per day, for a total exposure time of 190 and 380 h, respectively. The control group was reared in an animal room with an environmental background radon concentration of less than 40 Bq/m^3^. All of the rat groups had free activity, eating and drinking during the exposure.

The radon-exposure groups of rats were anesthetized with 10% chloral hydrate within 2–4 h after the last exposure. Blood specimens were collected from rat heart and used for PBLs isolation. Meanwhile, both sides of the femur were taken: one side for micronuclei of polychromatic erythrocytes (PCEMN) detection, and the other side for BMLs isolation and immunofluorescence assay.

### Immunofluorescence assay

The prepared PBLs and BMLs suspensions were adjusted to the cell density of ~6 × 10^5^ cells/mL each. The cells were adhered to glass slides pre-coated with a mixture of PLL and PLO using a cell centrifuge. The adherent cells were fixed with 4% paraformaldehyde for 15 min. The fixed cells were permeabilized with 0.5% (v/v) Triton X-100 in PBS for 15 min followed by blocking with 10% (v/v) fetal calf serum (FBS) in PBS at 37 °C for 1 h and then incubated for overnight at 4 °C with following primary antibodies all at a dilution of 1:500: rabbit polyclonal γ-H2AX (Ser-139) (Cell Signaling Technology, Danvers, MA, USA); mouse monoclonal p-ATM (Abcam (Hong Kong) Ltd., HK, China); mouse monoclonal p-DNA-PKcs (Abcam). After that, the cells were added with donkey anti-rabbit or donkey anti-mouse secondary antibody labeled with Alexa Fluor 488 or Alexa Fluor 555 (Molecular probe, Life Technologies, Grand Island, NY, USA) all at a 1:500 dilution and incubated at room temperature in the dark for 1 h. Each slide was dropwise added with DAPI-containing anti-fade reagent (Santa Cruz Biotechnology, Santa Cruz, CA, USA), covered with a coverslip, and mounted with nail polish. The slides were wrapped with aluminum foil and stored at −80 °C until used for microscopic observation.

### Foci counting under fluorescence microscopy

The numbers of green γ-H2AX foci tracks and γ-H2AX foci, along with red p-ATM foci and p-DNA-PKcs foci in the nucleus, were observed and scored manually under the Olympus BX51 fluorescence microscope (Tokyo, Japan). Images were captured using a COHU CCD camera (Audio Video Supply, San Diego, CA, USA) and VideoTesT-FISH software (VideoTesT, Saint-Petersburg, Russia). Overall, 3,000–8,000 cells were counted for each specimen to calculate the numbers of foci tracks and foci per cell. In addition, co-localization of γ-H2AX with p-ATM foci and with p-DNA-PKcs foci was examined. The co-localization ratios were calculated as follows: total number of co-localized foci ×2/total number of two types of foci. γ-H2AX foci tracks were divided into two types: a continuous linear fluorescent track formed by numerous foci, in which individual foci could not be distinguished; and a discontinuous linear track formed by morphologically distinguishable individual foci. When counting γ-H2AX foci, foci track in which individual foci could not be distinguished was counted as an elongated focus.

### Bone-marrow PCEMN detection

One side of the femur was taken, and BMLs were washed out with 0.5 mL of FBS. The cells were added with 1 mL of normal saline, mixed thoroughly, and then centrifuged at 1,000 rpm for 5 min. After the supernatant was discarded, the cell pellet was added with 0.5 mL of FBS and mixed by pipetting. The cell suspension was smeared, dried and fixed with 100% methanol. After Giemsa staining, microscopic examination was performed using oil-immersion lens. For each rat, the number of PCEMN in 1,000 polychromatic erythrocytes (PCEs) was counted. The frequency of PCEMN was obtained by dividing the number of PCEMN by the number of PCEs. Additionally, the numbers of PCEs and normochromatic erythrocytes (NCE) in 1,000 erythrocytes were counted to calculate the PCE/NCE ratio.

### Statistical analysis

The experimental data are provided as 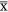 ± s. A statistical analysis was conducted using SPSS 20.0 (IBM SPSS, Somers, NY, USA). Comparison between two groups was completed using an independent sample *t* test. Multiple comparisons were carried out using one-way ANOVA. A *P* value of less than 0.05 was considered to indicate statistical significance.

## Additional Information

**How to cite this article**: Wang, J. *et al.* Establishment of a γ-H2AX foci-based assay to determine biological dose of radon to red bone marrow in rats. *Sci. Rep.* **6**, 30018; doi: 10.1038/srep30018 (2016).

## Figures and Tables

**Table 1:** Estimation of PBLs- and RBM- absorbed doses in rats inhaling radon and its progeny.

Tissue	Cumulative radon dose (WLM)	γ-H2AX foci/cell	Absorbed dose (mGy)	Absorbed dose ratio (RBM:PBLs)	Relative to the literature^46^ (0.0139 mGy/WLM)
PBLs	10	0.0010 ± 0.0001	—	—	—
	30	0.0031 ± 0.0004	0.2347 ± 0.0426	—	—
	60	0.0055 ± 0.0007	0.4990 ± 0.0741	—	—
RBM	14	0.0098 ± 0.0013	0.9747 ± 0.1548	—	4.99
	30	0.0188 ± 0.0023	2.0562 ± 0.2796	8.76	4.92
	60	0.0344 ± 0.0051	3.9418 ± 0.6135	7.90	4.71

Abbreviations: PBLs, peripheral blood lymphocytes; RBM, red bone marrow; WLM, working level month.

Data are presented as mean ± standard deviation.

**Table 2:** Damage changes in PBLs and BMLs of rats inhaling radon and its progeny.

Groups	No. of rats	Pan-nuclear γ-H2AX-positive cells (%)		PCEMN (%)	PCE/NCE
		PBLs	BMLs		
Control	10	0.58 ± 0.04	0.51 ± 0.05	0.81 ± 0.20	0.44 ± 0.08
10 WLM	10	0.61 ± 0.10	—	0.96 ± 0.45	0.44 ± 0.10
14 WLM	5	—	0.69 ± 0.10	—	—
30 WLM	6	0.65 ± 0.10	0.66 ± 0.09	0.98 ± 0.52	0.47 ± 0.10
60 WLM	6	0.82 ± 0.10	0.83 ± 0.25	2.78 ± 1.29*^#^	0.40 ± 0.06

Abbreviations: PCEMN, micronuclei of polychromatic erythrocytes; PCE, polychromatic erythrocytes; NCE, normochromatic erythrocytes.

Data are presented as mean ± standard deviation.

Compared with PCEMN (%) of control group, *P < 0.05; compared with PCEMN (%) of 30 WLM group, ^#^*P* < 0.05.
